# Mechanism of β-actin mRNA Recognition by ZBP1

**DOI:** 10.1016/j.celrep.2016.12.091

**Published:** 2017-01-31

**Authors:** Giuseppe Nicastro, Adela M. Candel, Michael Uhl, Alain Oregioni, David Hollingworth, Rolf Backofen, Stephen R. Martin, Andres Ramos

**Affiliations:** 1 Macromolecular Structure Laboratory, The Francis Crick Institute, London NW1 1AT, UK; 2 At the former MRC National Institute for Medical Research, Mill Hill, London; 3 Bioinformatics Group, Department of Computer Science, University of Freiburg, 79110 Freiburg, Germany; 4 MRC Biomedical NMR Centre, The Francis Crick Institute, London NW1 1AT, UK; 5 Mycobacterial Systems Biology Laboratory, The Francis Crick Institute, London NW1 1AT, UK; 6 Centre for Biological Signaling Studies (BIOSS), University of Freiburg, 79110 Freiburg, Germany; 7 Structural Biology Science Technology Platform, The Francis Crick Institute, London NW1 1AT, UK; 8 Institute of Structural and Molecular Biology, University College London, London WC1E 6XA, UK; 9 The Francis Crick Institute, London NW1 1AT, UK

**Keywords:** protein-RNA interactions, NMR, binding mechanism, neuronal mRNA granules, neuronal development, mRNA local translation, ZBP1, IMP1

## Abstract

Zipcode binding protein 1 (ZBP1) is an oncofetal RNA-binding protein that mediates the transport and local translation of *β-actin* mRNA by the KH3-KH4 di-domain, which is essential for neuronal development. The high-resolution structures of KH3-KH4 with their respective target sequences show that KH4 recognizes a non-canonical GGA sequence via an enlarged and dynamic hydrophobic groove, whereas KH3 binding to a core CA sequence occurs with low specificity. A data-informed kinetic simulation of the two-step binding reaction reveals that the overall reaction is driven by the second binding event and that the moderate affinities of the individual interactions favor RNA looping. Furthermore, the concentration of ZBP1, but not of the target RNA, modulates the interaction, which explains the functional significance of enhanced ZBP1 expression during embryonic development.

## Highlights

• The dynamic groove of ZBP1’s KH4 domain allows recognition of a G-rich RNA sequence
• ZBP1’s KH3 and KH4 domains bind their target RNA sequences with similar affinities
• RNA looping drives the ZBP1-*β-actin* interaction
• The protein, rather than the RNA, concentration regulates ZBP1-*β-actin* mRNA binding

## Graphical Abstract

**Figure:**
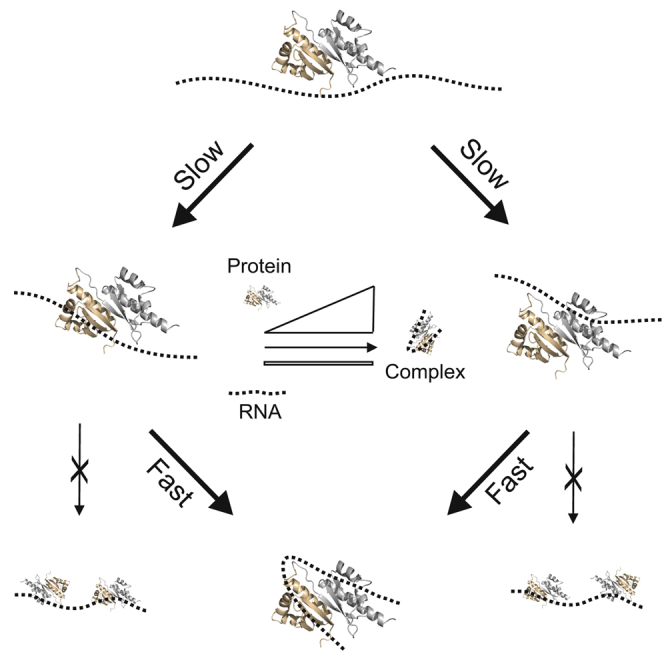

## Teaser

The interaction of *β-actin* mRNA with the RNA-binding protein ZBP1 regulates mRNA transport and translation and is necessary for neuronal development. Using NMR and biophysics, Nicastro et al. have shown that the interaction is driven by RNA looping and regulated by the protein, rather than the RNA, concentration.

## Introduction

Zipcode binding protein 1/IGF2 mRNA binding protein 1 (ZBP1/IGF2BP1/IMP1) is an oncofetal protein expressed at high levels in the embryo that is important for the development of the nervous system. A faulty protein or reduced ZBP1 gene expression results in impaired embryonic development (Hansen et al., 2004) and a smaller cerebral cortex (Nishino et al., 2013). At the cellular level, ZBP1 has been shown to be important for changes in cell proliferation, morphology, and motility (Conway et al., 2016, Katz et al., 2012, Farina et al., 2003, Vainer et al., 2008, Stöhr and Hüttelmaier, 2012, Maizels et al., 2015), and in developing neurons, ZBP1 regulates growth cone guidance, axonal remodeling, and dendritic morphology (Leung et al., 2006, Sasaki et al., 2010, Welshhans and Bassell, 2011, Medioni et al., 2014, Eom et al., 2003). In adults, ZBP1 expression is restricted to a small number of tissues and cells, but the protein is expressed at high levels in some cancers, which has been correlated with tumor growth and metastasis (Stöhr and Hüttelmaier, 2012, Bell et al., 2013).

**Figure 1:**
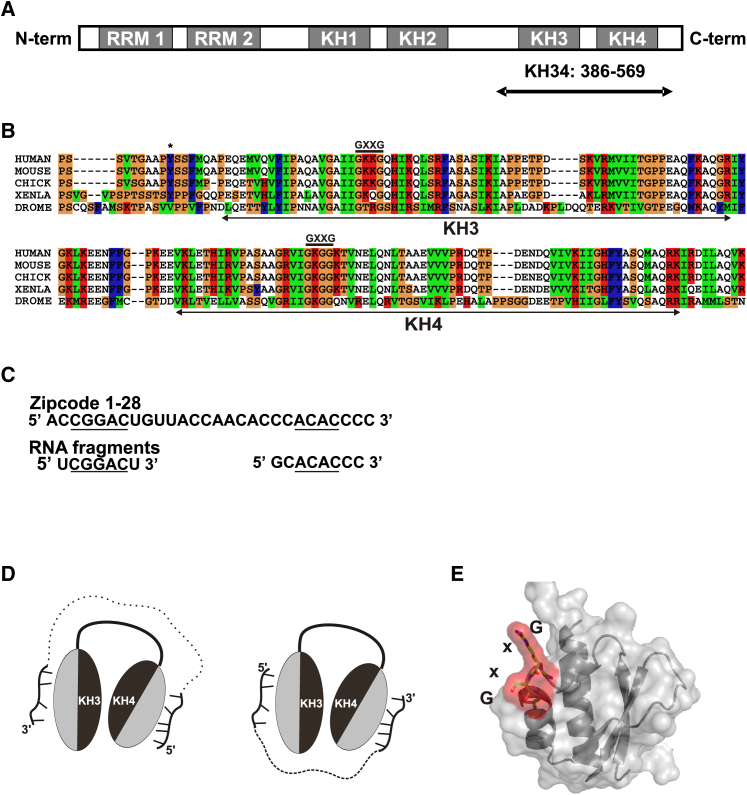
ZBP1 and *β-actin* mRNA (A) Domain organization of ZBP1 in vertebrates. The boundaries of the KH3-KH4 construct (chicken) are indicated by an arrow below the protein cartoon and include part of the KH2-KH3 linker. (B) Sequence alignment of KH3-KH4 domains in a human, mouse, chicken, frog, and *Drosophila* (Clustalx). Domain boundaries and the GXXG loops are highlighted. The residue phosphorylated by Src (Y395) is indicated by a star. (C) Top: chicken Zipcode 1–28 sequence as used (Patel et al., 2012). Bottom: RNA oligos used in the study. The KH3 and KH4 recognition motifs are underlined. (D) Cartoon representation of RNA looping around KH3-KH4. The two allowed orientations are shown. (E) KH domain RNA KO GxxG mutant (Hollingworth et al., 2012). Surface and ribbon representation of the domain (silver), with the GxxG residues displayed in red.

ZBP1 contains six putative RNA-binding domains (two RNA recognition motifs [RRMs] and four hnRNP K-homology [KH] domains) organized in three two-domain units, and is an RNA-binding protein (Nielsen et al., 1999, Yisraeli, 2005). ZBP1’s domain structure is conserved, except for the RRM domains, which differ in vertebrates compared to *Drosophila* (Nielsen et al., 1999, Yisraeli, 2005) (Figures 1A and 1B). Furthermore, the primary amino acid sequence of the individual RNA binding domains and the RNA sequence specificity of the well-studied KH3 and KH4 domains are also highly conserved (Farina et al., 2003, Patel et al., 2012). In the cell, ZBP1 interacts with a diverse range of mRNA targets (Conway et al., 2016, Patel et al., 2012, Jønson et al., 2007, Hafner et al., 2010, Hansen et al., 2015), and this interaction is important for the stability of the mRNA target and its transport and translational control (Leeds et al., 1997, Conway et al., 2016, Leung et al., 2006, Weidensdorfer et al., 2009, Hüttelmaier et al., 2005).

The functional importance of ZBP1 and the information available on its binding partners and mode of action has established this protein as a pivotal system to study mRNA transport and local translation during neuronal differentiation in the developing brain (Tolino et al., 2012). Equally important, the link between ZBP1 expression levels and tumor growth and metastasis (Stöhr and Hüttelmaier, 2012, Bell et al., 2013) identifies the protein as both a potential diagnostic tool (Bell et al., 2015) and a possible target for improving the outcome of lung and colon cancer (Maizels et al., 2015, Davidson et al., 2014). However, key molecular features of ZBP1-mediated regulation of its mRNA targets are not understood or have been described only qualitatively. A mechanistic and quantitative understanding of ZBP1-RNA interactions is vital to understanding how ZBP1 functions.

The best characterized mechanism mediated by ZBP1 is the regulation of the local translation of *β-actin* mRNA. ZBP1 associates with *β-actin* mRNA in the perinuclear space and mediates its transport in a translationally repressed form to the cell edge (Hüttelmaier et al., 2005). Once at the cell edge, ZBP1 is phosphorylated by Src in response to an extracellular signal, and the mRNA is released and translated (Hüttelmaier et al., 2005, Wu et al., 2015). The local increase in β-actin concentration favors actin polymerization and cellular remodeling and migration (Jung et al., 2014). At the molecular level, the RRM di-domain of ZBP1 interacts with the KIF11 molecular motor, which mediates the transport of the protein-RNA complex along the microtubules (Song et al., 2015). Furthermore, ZBP1 interaction with the *β-actin* mRNA is mediated by the two C-terminal KH domains of the protein, KH3 and KH4 (Farina et al., 2003, Patel et al., 2012), which recognize the *β-actin* 3′ UTR Zipcode RNA element (Figure 1C). The KH3 and KH4 domains are structurally linked to form an intra-molecular pseudo-dimer, with the two RNA-binding grooves on opposite sides. This arrangement implies that for a single RNA molecule to bind to both domains, it must loop around the protein (Figure 1D). The target sequences of KH3 and KH4 are separated by a spacer, and the length of this spacer is important for the interaction with the di-domain (Patel et al., 2012, Chao et al., 2010). In the *β-actin* Zipcode, the distance between the KH3 and KH4 target sequences is 14 nucleobases, whereas in other targets, the spacer length varies between 10 and 23 nucleotides. Interestingly, the 5′-to-3′ order of the KH4 and KH3 target sequences can be swapped, with only very minor changes in binding affinity in vitro (Patel et al., 2012). This creates a recognition unit, in which the RNA spacer can connect the sequences either 5′ to 3′ or 3′ to 5′ and run on either end of the di-domain unit without interacting with it (Figure 1D).

In this study, we use the well-characterized *β-actin* mRNA to analyze how the KH3 and KH4 domains of ZBP1 recognize their target sequences, what drives and limits the multi-step interaction, and how regulatory changes in the concentration of protein and RNA targets impact their interaction.

## Results

### Overall Structure of the KH3 and KH4 RNA Complexes

The interaction between ZBP1 KH3-KH4 and the *β-actin* Zipcode is the key event for *β-actin* mRNA recognition in the cell. However, the lack of molecular and structural information on KH3-KH4-Zipcode binding limits our mechanistic understanding of the interaction. The affinities of the individual KH3 and KH4 domains for the RNA targets are not known, and although the specificity of KH4 has been quantified in a recent study (Patel et al., 2012), that of KH3 has not, and it is unclear whether the results of recent RNA interactome studies (Conway et al., 2016, Hafner et al., 2010, Hansen et al., 2015) reflect a dominance of KH3 in target selection. Furthermore, we have no structural insight into how recognition occurs. We initially focused on the structural determinants of RNA recognition by KH3 and KH4. The RNA sequence linking the two motifs is very dynamic and does not contribute to the binding (Patel et al., 2012). Therefore, we could study the two mRNA target sites independently.

Starting from the Y396F mutant of the KH3-KH4 construct (referred to hereafter as KH3-KH4), we knocked out the ability of each individual domain to bind its RNA target and studied RNA binding by the other domain. The Y396F mutation leads to constitutive RNA binding by removing the effect of Src phosphorylation (Hüttelmaier et al., 2005). The mutant, used here for practical reasons over the wild type, binds RNA as the non-phosphorylated wild-type protein in the cell (Wu et al., 2015) and has no effect on *β-actin* mRNA localization in growth cones and therefore on functional RNA binding (Sasaki et al., 2010). In our in vitro system, RNA-binding knockout was achieved by mutating the two variable amino acids within the conserved GxxG loop to D. This mutation prevents interaction with the RNA backbone and eliminates any detectable RNA binding at near millimolar protein and RNA concentration without affecting the structure or stability of the domain (Hollingworth et al., 2012) (Figure 1E), as we recently showed for the KH domains of different proteins, including the ZBP1 KH3 and KH4.

**Figure 2:**
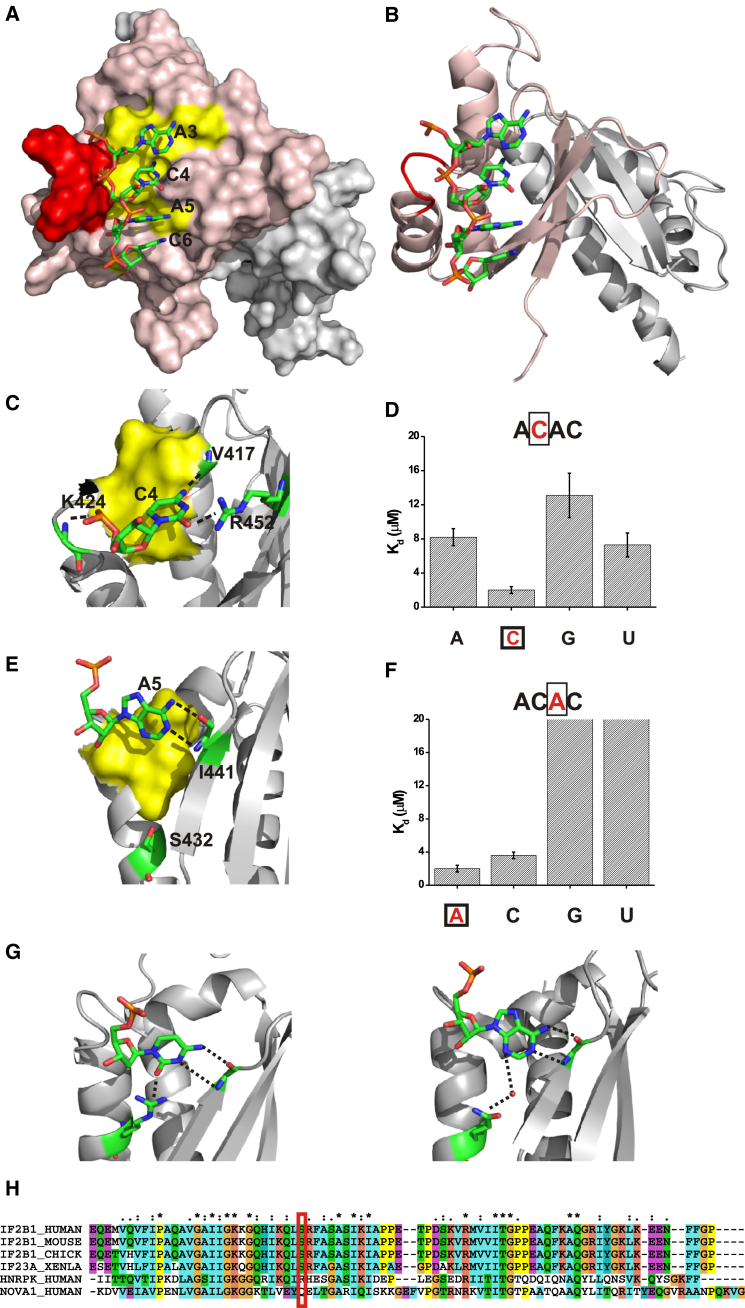
Structure and Specificity of the KH3-RNA Complex (A) The KH3-RNA complex. Surface representation of the bound KH3-KH4DD protein and stick representation of the cognate ACAC sequence. The RNA is colored by atom type, whereas the KH3 protein surface is pink, except for the GxxG loop (red) and the hydrophobic groove (yellow). The KH4 protein, in the back, is colored in gray. (B) The same complex is represented using a cartoon for the protein. KH3 is in pink, KH4 in grey. (C) Detail of the structure: the C4-KH3 interaction. Cartoon representation of the bound KH3 secondary structure. The KH3 hydrophobic surface contacting the RNA is in yellow, whereas the residues H-bonded to the RNA and the RNA itself are displayed using a stick representation. (D) K_ds_ of the protein in complex with, from left to right, the CAAAC, CACAC (wild type [wt]), CAGAC, and CAUAC RNAs. K_ds_ were measured using ITC. Raw and fitted data can be found in Figure S4, together with more experimental details. K_d_ values are represented as a histogram and are capped at 20 μM in the figure to represent the approximate limit at which an accurate figure can be obtained. Data fitting error is reported. All experiments were repeated twice. (E) Detail of the structure: the A5-KH3 interaction. Color coding and representation as in (C). (F) K_d_s of the protein in complex with, left to right, CACAC (wt), CACCC, CACGC, and CACUC. ITC experiments were performed and analyzed as in (D). (G) Comparison of the position 3 nucleobase H-bonding in the hnRNP K KH3-RNA (left) and Nova KH3-RNA (right). Regardless of the identity of the nucleobase, a third H-bond is observed with equivalent residues in helix 2, which is either an R (for C) or a Q (for A). (H) Alignment of the ZBP1 KH3 sequences in vertebrates with the Nova-1 KH3 and hnRNPK sequences (ClustalX). The residue in helix 2 H-bonded to A5 is boxed in red. See also Figures S1–S5.

**Figure 3:**
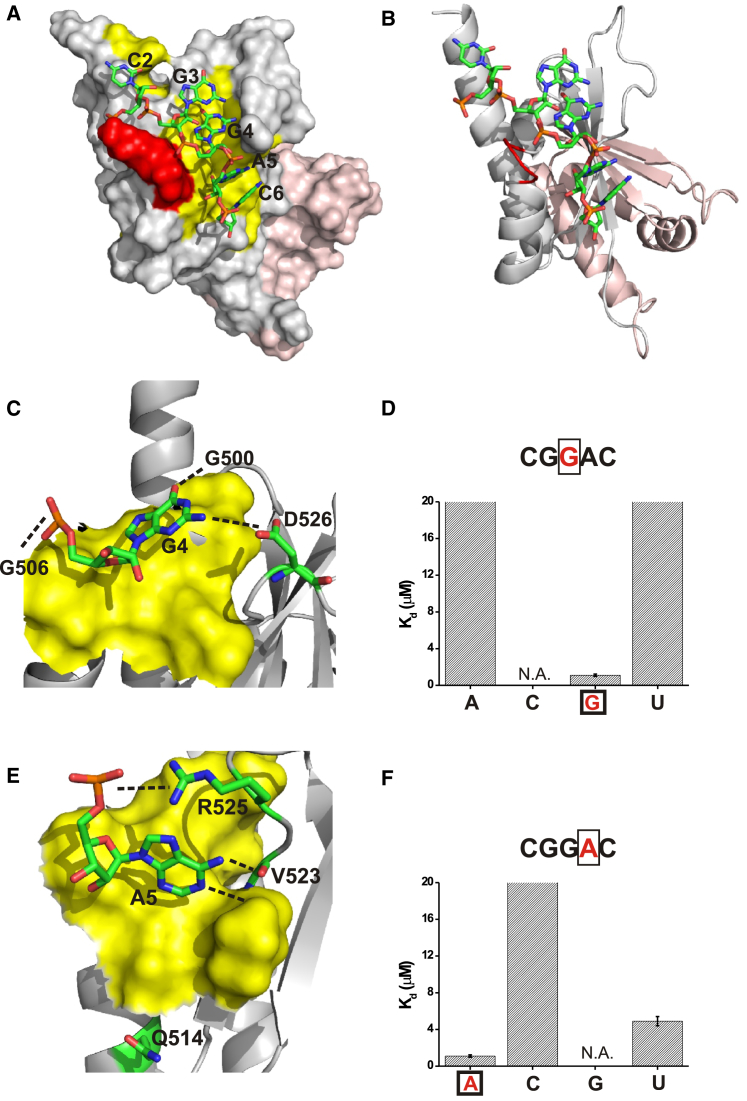
Structure and Specificity of the KH4-RNA Complex (A) The KH4-RNA complex. Surface representation of the bound KH3DD-KH4 protein and stick representation of the cognate CGGAC RNA nucleotides. The RNA is colored by atom type, whereas the KH4 protein surface is gray, except for the GxxG loop (red) and the hydrophobic groove (yellow). The KH3 protein, in the back in this orientation, is colored in pink. (B) The same complex is represented using a cartoon for the protein. KH4 is in grey, KH3 in pink. (C) Detail of the structure: the G4-KH4 interaction. Cartoon representation of the bound KH4 secondary structure. The KH4 hydrophobic surface contacting the RNA is in yellow, whereas the residues H-bonded to the RNA and the RNA itself are displayed using a stick representation. (D) K_d_s of the protein in complex with, from left to right, the UCGAACU, UCGGACU (wt), and UCGUACU RNAs. K_d_s were measured using ITC. Raw and fitted data can be found in Figure S4, together with more experimental details. K_d_ values are represented as a histogram and are capped at 20 μM in the figure to represent the approximate limit at which an accurate figure can be obtained. Data fitting error is reported, and all experiments were repeated twice. We attempted to measure the affinity of the protein for the UCGCACU RNA but could not obtain reliable K_d_ measurements (N.A., not available). (E) Detail of the structure, the A5-KH4 interaction, which is color coded as in (C). Please note that a water-mediated H-bond is likely to exist between Q514 and A5 based on distance, geometry, and similarity with other structures, but cannot be directly detected using NMR. (F) K_d_s of the protein in complex with, from left to right, the UCGGACU (wt), UCGGCCU, and UCGGUCU RNAs. K_d_s were measured using ITC as described in (D). The UCGGGCU formed G-quartet structures and did not provide reliable K_d_ measurements (N.A., not available) in the third column. See also Figures S1–S5.

Using the two mutants and nuclear magnetic resonance (NMR) spectroscopy (Figure S1), we solved the solution structures of the KH3-KH4DD (KH4 KO) protein bound to the target GCACACCC RNA and of KH3DD-KH4 (KH3 KO) RNA bound to the UCGGACU RNA (KH3 and KH4 recognition motifs [Patel et al., 2012] are underlined) (Figures 2A, 2B, 3A, and 3B), which recapitulate the contacts made between KH3-KH4 and the Zipcode RNA (Figures 1C and S2; Table 1). In both the KH3-KH4DD-RNA (KH3 binding, KH4 KO) and KH3DD-KH4-RNA (KH3 KO, KH4 binding) complexes, the bases orient toward the hydrophobic groove, where the Watson-Crick edges are recognized by a network of hydrophobic interactions and H-bonds (Figures 2C, 2E, 3C, 3E, and S3). In both structures, the interacting nucleotides have sugars in a 3′ *endo* conformation, whereas the glycosidic angle is in an *anti* conformation (Figures 2A, 2B, 3A, and 3B). Beyond this general KH-RNA binding mode (Nicastro et al., 2015), the structural analysis identifies features that have not been reported in KH-RNA recognition and that relate to RNA recognition by the individual KH3 and KH4 domains and to their different binding kinetics. We describe the most important of these features below.

### KH3 and KH4 Recognize RNA with Very Different Specificity

Recently published SELEX data indicate that KH3 has an absolute sequence preference for a CA dinucleotide in the central position of the C/UCAC/A four-nucleotide recognition sequence (Patel et al., 2012), although, importantly, mutation of two As within the *β-actin* Zipcode KH3 RNA target was shown to lead to only a few-fold change in affinity in the same study. We find that in the KH3-RNA complex, the two central nucleobases (C4 and A5) are recognized via multiple H-bonds and hydrophobic contacts (Figures 2C, 2E, and S3). In our structure, two H-bonds are formed between the Watson-Crick edge of the C4 base and two amino acids in the KH3 β sheet and variable loop, V417 and R452, respectively. However, the difference in affinity between C and the other three nucleobases is significantly lower than what has been reported for the equivalent position in other KH-RNA interactions, suggesting a lower sequence specificity (Nicastro et al., 2015, Jensen et al., 2000, Backe et al., 2005). Isothermal titration calorimetry (ITC) measurements (Figures 2D and S4) show that mutation of C4 to any other nucleotide leads to weaker binding (4- to 7-fold higher K_d_), but the energy penalty for binding a different nucleobase in this position is much lower than that reported for Nova-1 KH3 and other KH domains (Nicastro et al., 2015, Jensen et al., 2000). Furthermore, we observed that A5 is recognized with high specificity with respect to G or U (20-fold affinity difference), but not with respect to C (2- to 3-fold affinity difference) (Figures 2F and S4). This weak A/C discrimination is much lower than that reported for other canonical KH-RNA interactions (Jensen et al., 2000, Backe et al., 2005), in which differences in affinity can reach more than 50-fold, further indicating that ZBP1 KH3-RNA binding occurs with low specificity.

Comparison of the inter-molecular contacts in the ZBP1 KH3-RNA complex with the contacts in the published KH-RNA and KH-DNA structures (Nicastro et al., 2015) explains the low C/A discrimination in KH3. Canonical A/C versus G/U recognition by a KH domain involves a double H-bond between the Watson-Crick edge of the base and an amide and carboxy group in the protein backbone, which, in the ZBP1 KH3-RNA complex, is formed between A5 and the backbone moieties of I441 (Figure 2G). Furthermore, a third H-bond, either direct or water mediated, is normally observed between the Watson-Crick edge of the nucleobase and an amino acid (either Gln or Arg) in the second alpha helix of the KH domain (Figure 2G). We propose that the nature of the amino acid defines A versus C selectivity in this position. A Gln residue forms a water-mediated H-bond with the N3 moiety of an A, whereas recognition of a C is mediated by an H-bond between the CO2 and the guanidium group of an Arg residue in canonical KH-RNA recognition (Nicastro et al., 2015) (Figure 2G). In ZBP1, the corresponding amino acid is a Ser residue (S432). The distance from the S432 side chain OH oxygen to the edge of the nucleobase (∼8 Å, Figure 2E) is such that no direct or water-mediated H-bond can form. The equivalent distance in Nova-1 KH3, where a water-mediated H-bond exists, is 5.8 Å. A serine is not observed in other KH-RNA complexes, but is conserved in ZBP1, where its side chain does not, however, engage in structural contacts, highlighting that this feature is both unique to ZBP1 KH3 and functionally important, as discussed below (Figure 2H).

In contrast to KH3, our structure and ITC assays on the KH4-RNA complex show that the large G nucleobases (G3 and G4) are inserted in a non-canonical hydrophobic groove that is unusually large and open, and strong nucleobase discrimination is mediated by a combination of hydrophobic interactions and H-bonds (Figures 3C–3F and S3). By contrast, recognition of an adenine (A5) in this position of the target sequence represents a common choice for KH domains. Furthermore, in contrast to KH3, A/C discrimination in this position is very strong, as measured by a greater than 20-fold difference by ITC (Figures 3E and 3F). Indeed, we observed that both the canonical double H-bond with the protein backbone and the third H-bond with a Gln residue are present in the structure (Figures 3E and S3). However, and consistent with a previous report (Patel et al., 2012), KH4 discriminates much less strongly (4-fold only in our ITC measurements) against a U (Figures 3 and S4). There is no obvious single amino acid substitution that can explain this, and in silico modeling followed by energy minimization suggests that the shape and size of the groove allows a rearrangement of the nucleobase position to create alternative H-bonds, possibly between the U carbonyl C4 and R525 guanidinium groups and between the Val 523 backbone and the U N3 (data not shown). Nucleobases 5′ and 3′ to the central GGA sequence are also bound specifically, as previously described using electrophoretic mobility shift assays (EMSAs) (Patel et al., 2012).

Importantly, the high-sequence specificity of KH4 with respect to KH3 and the similar binding affinities of the two domains (KH4 K_d_ ∼1.5 μM, KH3 K_d_ ∼2 μM, Figure S4) indicate that although the KH4 interaction is significantly more specific than the KH3 interaction, both domains are likely to contribute to binding.

### KH4 Associates and Dissociates with the RNA Faster Than KH3 and Has a More Dynamic RNA-Binding Groove

**Figure 4:**
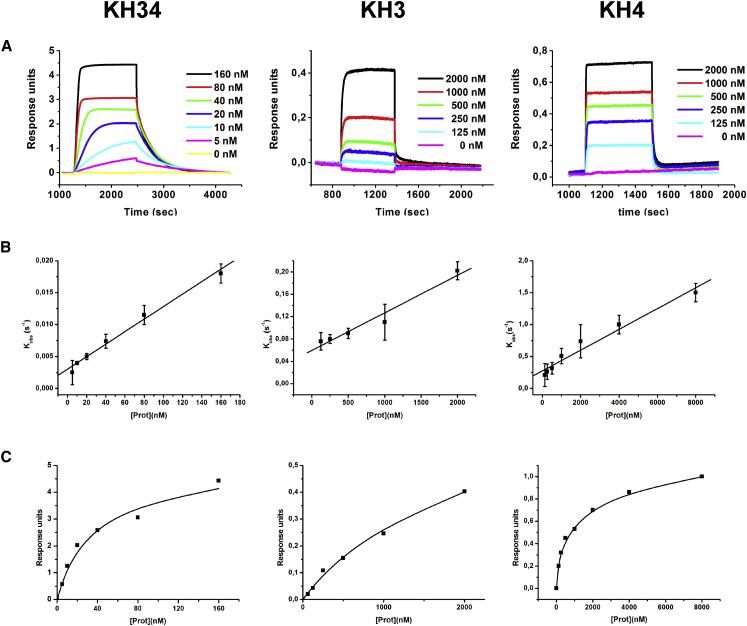
Interaction of KH3-KH4, KH3, and KH4 with the *β-actin* Zipcode RNA by BLI (A) Response of Streptavidin-coated sensors derivatized with biotinylated Zipcode RNA and exposed to increasing concentrations of KH3-KH4 (left), KH3-KH4DD (middle), and KH3DD-KH4 (right). Data are aligned using the baseline step, and the baseline, association, and dissociation step are displayed. (B and C) k_obs_ plotted against protein concentration (B) and response plotted against protein concentration for the same experiments (C).

Binding of KH3 and KH4 to their respective target sequences is coupled in vitro and in the cell, and KH3-KH4 inter-domain coupling is essential for interaction with the *β-actin* mRNA (Patel et al., 2012). To dissect this coupling and understand the role of the two domains in the interaction, we first measured both affinity and kinetic parameters for the interactions of individual domains within the two-domain structure, then related them to the dynamics existing in the two RNA-binding grooves and the structural context of the RNA targets, and finally explored how the domains cooperate to bind the Zipcode RNA (Figures 4 and S5).

We recorded biolayer interferometry (BLI) experiments on an immobilized 28-nucleotide Zipcode RNA exposed to different concentrations of ZBP1 KH3DD-KH4 (KH3 KO), KH3-KH4DD (KH4 KO), or KH3-KH4 (Figure 4), and obtained equilibrium dissociation constants (K_d_) as well as kinetic parameters (k_on_ and k_off_) for the interaction. The equilibrium dissociation constants for the Zipcode RNA-KH4 KO and KH3 KO complexes are 1.5 μM and 0.9 μM, respectively. This is comparable with the K_d_s we measured for the same two protein constructs in complex with the short RNA target sequences using ITC, which confirms that these interactions recapitulate those with the full-length Zipcode (Figures 4 and S4). Interestingly, although the affinities of the two domains are similar, the kinetic constants are different. KH4 associates with the RNA target five times faster than KH3 (k_on_: 1.4 × 10^5^ M^−1^s^−1^ versus 3.0 × 10^4^ M^−1^s^−1^). Conversely, the dissociation rate of the KH3-RNA complex is three times slower than that for KH4 (k_off_: 0.13 s^−1^ versus 0.046 s^−1^).

With the exceptions of the flexible amino and carboxy-terminal regions flanking the di-domain, the motions are generally limited in the structure (Figure S5). An exception is the variable loop of the bound KH4, which is less well defined in the structure of the complex (Figure S2), consistent with the low number of nuclear overhauser effect (NOE) cross peaks (distance correlations) observed in the KH4-bound groove. This hints at a more dynamic RNA-binding surface, and examination of the backbone motions taking place in the KH4 domain by NMR spectroscopy shows that a number of amino acids in that surface have lower heteronuclear NOE values (residues K505, T509, N511, Q514, A519, V521, and E533) or higher T2 values (residues R525 and Q527), likely stemming from high-frequency motions often observed in flexible regions (Figure S5). A similar pattern of NOE and T2 values is observed in the RNA-bound KH4. However, no such motions are observed in the groove of the KH3 domain. Although different dynamic phenomena have been reported in the variable loop and in general in the hydrophobic groove for some KH domains, this region is normally locked by the binding of the nucleic acid target. Counterintuitively, and unique among KH domains as far as we are aware, the highly specific ZBP1 KH4 domain shows a significant degree of freedom in the RNA-interacting groove, which is maintained upon RNA binding. Finally, it is worth mentioning that a preliminary bioinformatics analysis using the GraphProt program (Maticzka et al., 2014) (data not shown) suggests that in the ensemble of putative KH4-binding sites, the KH4 sites are more likely to engage in structural contacts than the KH3 ones, but are unlikely to form stable and conserved structures.

### KH3 and KH4-RNA Interactions Are Weakly Coupled

Having assessed the individual domain interactions with the Zipcode RNA sequence, we analyzed the interaction of this RNA with a construct in which both domains can engage in the interaction. KH3-KH4 binds to the RNA with a K_d_ of 20 nM, indicating that the coupling of KH3 and KH4 binding increases the affinity of the individual interactions by a factor of ∼50. Although the 20 nM K_d_ is higher than that previously reported based on EMSA assays (∼4 nM) (Patel et al., 2012), the difference may be explained in large part by the lower temperature of the EMSA assays (5°C versus 25°C for the BLI). Indeed, ITC data recorded at 25°C (Figure S4) confirmed that the binding affinity of KH3-KH4 for the Zipcode sequence is close to the 25°C BLI values.

Analysis of the kinetics of the KH3-KH4 interaction revealed that the association rate constant for KH3-KH4 is very similar to that for the KH4-RNA interaction (1.6 × 10^5^ M^−1^s^−1^ versus 1.4 × 10^5^ M^−1^s^−1^). In contrast, the dissociation rate constant for KH3-KH4 (0.0033 s^−1^) is between one and two orders of magnitude lower than that of either mutant, indicating that the higher affinity of the KH3-KH4 interaction with Zipcode RNA results almost entirely from the lower dissociation rate. The relatively weak interaction of the individual domains with RNA (∼1 μM) and the weak coupling of KH3 and KH4 binding (one to two orders of magnitude) is similar to that observed for a number of other multi-domain RNA-binding proteins and has been proposed to respond more readily to regulation than a single high-affinity interaction (Lunde et al., 2007, Mackereth and Sattler, 2012).

### ZBP1-RNA Interaction Is Driven by the Looping of the Target RNA

**Figure 5:**
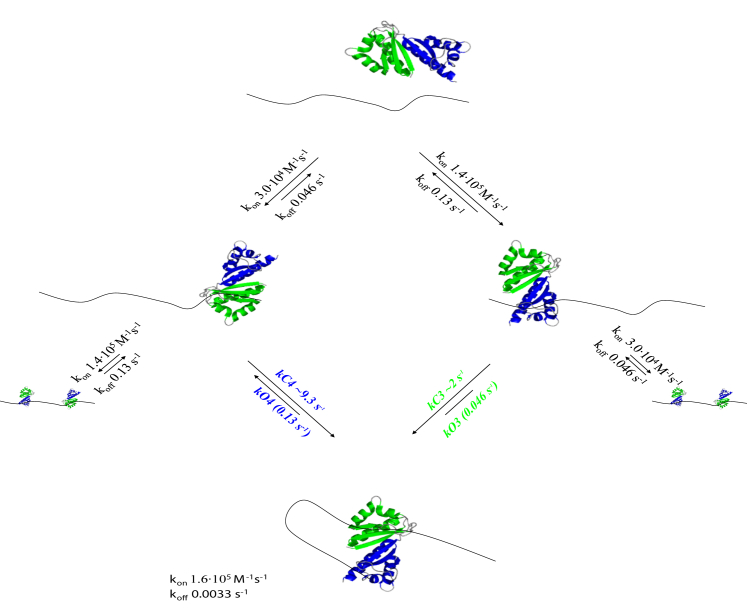
Kinetic Model for the KH34 Interaction The association and dissociation rate constants were determined using BLI, as described in the main text. The RNA is represented by a black line, whereas the KH3 and KH4 protein domains are represented using a secondary structure cartoon built from the free protein coordinates. In our model, either domain of KH3-KH4 can associate with its cognate sequence on the Zipcode to form a 1:1 complex. Each of the two possible complexes formed in this way can then proceed through a “ring-closure” step, in which the remaining unbound domain binds to its cognate RNA sequence. Alternatively, a second KH3-KH4 protein can bind to the unoccupied cognate sequence. The second scenario leads to the formation of a 2:1 protein-RNA complex, whereas the first leads to RNA remodeling. The rate constants for the ring-closure event (which we name closing constants for KH3 and KH4 or kC3 and kC4) depend on both the speed at which the unfolded RNA can explore the conformational space and the time required to make a productive contact with the second domain once it is in proximity to its binding site, as detailed in the Supplemental Experimental Procedures section.

The binding of KH3 and KH4 to the Zipcode mRNA can be described in a kinetic simulation that provides insight into the KH3-KH4-RNA interaction and RNA remodeling at a mechanistic level (Figure 5). Building the simulation allows a quantitative description of the kinetic pathways over time and how changes in the levels of free and bound protein and RNA species impacts ZBP1 binding and functional output. The simulation can also be used to derive information on timescales that are not experimentally accessible, i.e., the one of the second binding event.

In the KH3-KH4-RNA interaction, a first binding event by either KH3 or KH4 is followed by binding of the second domain and looping or remodeling of the RNA (Figure 5). Alternatively, a second protein could bind to the same RNA (2:1 protein:RNA complex). Association and dissociation rate constants for the individual domain and di-domain interactions were obtained using BLI (see above). A simulation based on these rate constants and published estimates of cellular protein and RNA concentrations (Wu et al., 2015, Buxbaum et al., 2014, Batish et al., 2012) can then be used to estimate the rate(s) for the second protein domain binding to the same RNA (which involves RNA looping). We define these as closing rates kC3 and kC4, depending on which domain is involved in the second binding event (see Figure 5).

**Figure 6:**
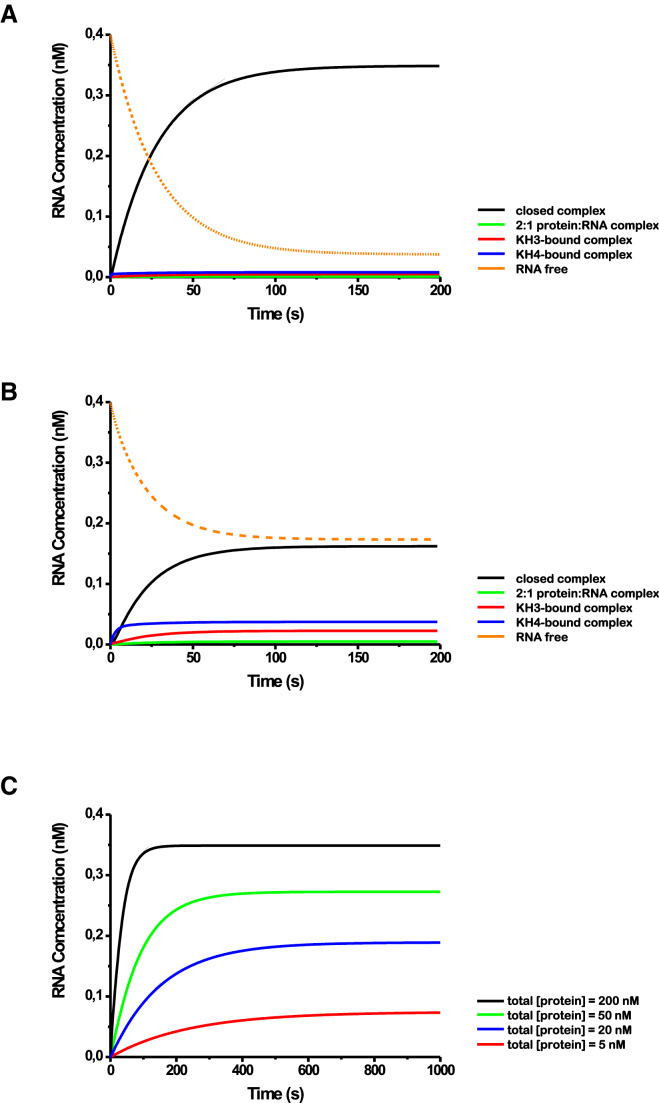
Simulations of the Interaction between IMP1 KH3-KH4 and Zipcode RNA (A and B) Simulations reporting on the concentration of the different protein-bound RNA species during the time course at kC3 = 2 s^−1^(A) and 0.2 s^−1^ (B), which correspond to K_d_s of ∼20 and ∼150 nM, respectively. Free RNA (orange, dotted line), closed complex (black), 2:1 protein:RNA open (green) complex, KH3-bound (red) complex, and KH4-bound (blue) complex are plotted. In both cases, the closed complex is the dominant species, although at a lower kC3 value, the concentration of single-domain bound species is no longer negligible. The 2:1 open complex concentration is negligible, regardless of kC3. Equilibrium is reached between 100 and 150 s, regardless of the values of kC3 and kC4. (C) Simulations reporting on the concentration of closed complex with kC3 of ∼2 s^−1^ for protein concentrations of 5 (red), 20 (blue), 50 (green), and 200 (black) nM. Both the total amount of bound protein and the association rate strongly depend on the concentration of the protein.

Precise cellular concentrations of protein and RNA are difficult to obtain, but recent independent studies have estimated ∼500 molecules of *β-actin* mRNA to be present in primary neurons (Buxbaum et al., 2014, Batish et al., 2012). In a 20- to 40-μm diameter hemispherical-shaped cell, this corresponds to a subnanomolar mRNA concentration. On the other hand, recent work has shown that the ZBP1 concentration in a population of mouse embryonic fibroblasts (MEFs) ranges from 0.05 to 0.5 μM (Wu et al., 2015). We therefore used protein and RNA concentrations of 0.2 μM and 0.4 nM (as estimates), respectively, in our simulations. Although the kinetic rate constants of KH3 and KH4 binding to the RNA zipcode can be measured using BLI, the closing rates kC3 and kC4 are not experimentally accessible. Instead, values for kC3 (∼2 s^−1^) and kC4 (∼9.4 s^−1^) were derived from the measured K_d_ of the KH3-KH4-RNA complex and the on- and off-rate constants of the individual binding events, as detailed in the Supplemental Experimental Procedures. We then used in-house developed software (fourth-order Runge-Kutta method, see the Experimental Procedures section) and the two calculated kC values to compute the time courses for the different binding events. As a first step, a computer simulation was run using kC3 = 2 s^−1^, kC4 = 9.333 s^−1^, [KH3-KH4] = 0.2 μM, and [RNA] = 0.4 nM until equilibrium was reached, and the K_d_ was calculated from the concentration of the appropriate species (Figure 6). The resulting K_d_ (20.6 nM) was in close agreement with the experimentally measured value (20 nM), as was the dissociation rate (0.0034 s^−1^ calculated versus 0.0033 s^−1^ experimental, Figure 4), which validates the computational procedure as an accurate and useful tool.

This analysis also shows that a first slower binding event that depends on protein concentration is followed by the fast concentration-independent binding of the second domain in the two-domain unit that drives the overall reaction toward formation of the closed complex. The alternative binding pathway, i.e., binding of a second protein to the same RNA, would require a significantly higher affinity for the two interactions because the cellular concentrations of protein and RNA are low compared with the K_d_s of the individual domains. Binding of a second RNA to the unoccupied domain of a 1:1 protein RNA complex to create a 1:2 complex is therefore not relevant in our analysis. Indeed, even if the closed complex did not form at all, very little of the double complex would be formed. A simulation performed under standard conditions (200 nM protein and 0.4 nM RNA), but with no closed complex formation, reached equilibrium with 38 pM 1:1 complex with KH3 bound, 63 pM 1:1 complex with KH4 bound, and only 8.2 pM double complex.

Although this simulation provides us with an assessment of the reaction time course, it is important to understand how variation in the kCs (for example, due to differences in the length of the spacer between the target RNA sequences) would impact this time course. That is, these simulations are important to establish to which extent a larger or smaller kC3 or kC4 (for example, due to an increased distance between the KH3 and KH4 target sites) would affect the overall interaction. First, we explored the effect of varying the kC value on the behavior of the system by calculating K_d_ values, fractions of RNA bound at equilibrium, and dissociation rates (Table S1). The result shows that we expect little change in the percentage of bound RNA when kC3 values are increased. However, reducing kC3 values leads to a significant increase in the K_d_ and a corresponding decrease in the percentage of bound RNA (from ∼91% to ∼57% with kC3 reduced by an order of magnitude to 0.2 s^−1^). The simulations also show that although a smaller closing rate would lead to an increased K_d_ and a correspondingly lower fraction of bound RNA, the fraction of RNA present as 2:1 protein to RNA complex remains negligible, i.e., most of the mRNAs would still be looped and bound by both domains.

### ZBP1-RNA Interaction Is Regulated by Protein, not RNA, Concentration

An understanding of ZBP1 regulation during neuronal development requires an analysis of how the system would respond to changes in the concentration of protein and RNA. Protein concentration is in large excess (approximately three orders of magnitude) in the cell, and the fraction of ZBP1-Zipcode RNA bound is largely independent of the concentration of the *β-actin* mRNA. Indeed, our calculations show that were the cellular concentrations of the *β-actin* mRNA to be several fold higher or lower than our estimate, this would have no significant impact on what fraction of the *β-actin* mRNA is predicted to be bound by ZBP1. In contrast, the fraction of *β-actin* mRNA bound by ZBP1 would readily respond to changes in the concentration of the protein, indicating that the ZBP1 concentration controls the interaction. At a KH3-KH4 concentration of ∼5 nM, the fraction of bound RNA is 0.19, whereas at ∼50 nM, it is 0.71, and at ∼200 nM, it is 0.91 (Figure 6C; Table S2). Additionally, changes in the ZBP1 concentration regulate the speed of binding (Figure 6C), resulting in a very effective regulation of the protein-RNA interaction.

The regulation of protein-RNA interactions is a complex multi-factorial phenomenon, and *in cell* quantitative data on protein and RNA concentrations and binding affinities are not copious. However, recent microscopy data obtained on the ZBP1-*β-actin* mRNA interaction have provided an estimate of both the protein concentration and, importantly, the average number of protein molecules bound to each RNA molecule (i.e., the fraction of bound RNA because only one binding site is present in the RNA molecule [Patel et al., 2012]) in fibroblasts and hippocampal neurons [Wu et al., 2015]). This allowed us to compare the findings derived from our simulation to these cellular data. Looking at the ensemble of cells examined by Wu and colleagues (Figure 2B of Wu et al., 2015), we observed that the fraction of bound *β-actin* mRNA is dependent on the concentration of ZBP1, between a concentration of 0.05 and 0.4 μM. Our model predicts that at a 0.05-μM concentration, 71% of mRNA is bound, whereas at a 0.1-μM protein concentration, 83% protein is bound, and at a 0.2-μM protein concentration, 91% protein is bound (Table S2). It is not possible to precisely compare the values derived for our in vitro system with the fraction of bound RNA measured in the cell because the number of cells is limited and the variability is high. Nevertheless, we expect the in-vitro-measured K_d_ to be only a few fold lower than the K_d_ required to obtain the concentration-dependent *in cell* trend described above, which is well within the range we modeled (Table S1). This small difference can, in part, be explained by the lower temperature of our in vitro measurements (25°C versus 37°C in the cell), and some difference is, in general, not unexpected. Overall, our in vitro BLI data on the strength of the ZBP1-*β-actin* interaction are remarkably consistent with data measured in mouse embryonic fibroblasts and hippocampal neurons and validate the relevance of the KH3-KH4-Zipcode interaction in guiding ZBP1-*β actin* mRNA association in the cell. Further, the range of K_d_s examined in our simulations (Table S1) encompasses the difference between in vitro and *in cell* estimated K_d_ values and indicates that our mechanistic conclusions would hold in this range.

## Discussion

The role of ZBP1 in the cellular transport of *β-actin* mRNA is the best studied function of this protein and an important example of how RNA-binding proteins regulate local protein translation in neuronal development (Doyle and Kiebler, 2011). ZBP1 KH3 and KH4 domains are the key recognition elements for *β-actin* mRNA, but we show they bind their respective RNA targets very differently. On the one hand, KH4 recognizes its target RNA with a high degree of specificity. On the other hand, KH3 recognizes a shorter RNA sequence with lower specificity. KH3 binds with similar affinity to sequences containing CC and CA in their central positions, which is unusual because these positions are normally strongly defined. We could attribute the weak CC/CA discrimination of KH3 to the absence of an H-bond observed in other KH-RNA structures and show that the amino acid responsible is conserved in ZBP1 KH3 (but not in other KH domains) and has a solvent-exposed side chain that faces the RNA (Figure 2). The selective pressure to conserve this amino acid is arguably connected to the general RNA-binding properties of the domain, and because bioinformatics analysis shows that the KH3 is found in AC-rich regions with low structural content (Maticzka et al., 2014), it is tempting to speculate that the resulting weak A/C discrimination would facilitate KH3 transiently binding (“scanning”) the C-rich sequence surrounding the CA-recognition site in the *β-actin* Zipcode (Figure 1C) and other target mRNAs.

Interestingly, the short-binding motifs for full-length ZBP1 identified in two independent cross-linking and immun precipitation (CLIP) assays (Conway et al., 2016, Hafner et al., 2010) contain a CA sequence, and a CA di-nucleotide is part of the KH3 (but not of the KH4) target sequence. However, the CLIP-derived sequence preference for a CA dinucleotide reflects the overall contributions of all the RNA-binding domains of ZBP1 (Wächter et al., 2013). The similar binding affinities of KH3 and KH4, together with our computational simulations, suggest that both domains contribute to the recruitment of the RNA to the protein, with KH4 being most important for sequence specificity, as discussed above. Based on this, and considering that KH3 and KH4 act as a di-domain unit, it is unlikely that the strength of the CA signal in previous iCLIP data exclusively reflects a dominant contribution of KH3 but instead could encompass also the contributions of one or more of the RRM1 and 2 and KH1 and 2 domain, for which no sequence specificity is available.

Crucially, KH3 and KH4 bind in a coupled fashion. The two individual domains each have moderate RNA-binding affinities, and coupling of the two interactions is necessary for the interaction in vivo (Patel et al., 2012). However, we show that the coupling is relatively weak. The two-domain unit binds with an affinity ∼50-fold higher than that of the individual domains. This low level of coupling has been observed in other multi-domain nucleic-acid-binding proteins, such as PTB, KSRP, and many others (Lunde et al., 2007, Mackereth and Sattler, 2012), and is thought to facilitate regulation of the interaction. Our simulation indicates that at the relatively low cellular ZBP1 protein concentration, the moderate binding affinity of the individual domains would prevent non-stoichiometric RNA binding, instead favoring RNA looping, which is not dependent on the ZBP1 concentration.

RNA looping has been reported for other RNA-binding proteins, including the alternative splicing regulator PTB (Oberstrass et al., 2005, Auweter et al., 2007, Lamichhane et al., 2010), which is present at similar concentration as ZBP1 in cancer cells (Beck et al., 2011) and where the individual RRM3 and RRM4 domains bind RNA with moderate affinity (K_d_ in the low μM range; Auweter et al., 2007) and an ∼90-fold inter-domain coupling. It seems probable that the key quantitative insights we discussed above also apply to the PTB system.

In addition to providing information on the forces driving RNA looping, our model gives unique insight into the timescale of RNA looping, which, as far as we are aware, has not been experimentally characterized in ZBP1, PTB, or any other structurally equivalent systems. The simulations show that the looping associated with the binding of the second domain to the RNA takes place in less than a second after the formation of the first concentration-dependent complex. This includes both the time the RNA must spend exploring the conformational space around the di-domain to reach the proximity of the second hydrophobic groove to be bound and the time required for a productive interaction when the protein groove and the RNA cognate sequence are in physical proximity. It is worth considering that several assembly and RNA remodeling steps are likely to be required to build a ZBP1-*β-actin*-containing ribonucleoprotein particle, and the overall time of assembly is likely to be significantly slower. As more kinetic data become available on inter-coupling in protein-RNA interactions, we expect it will be possible to explore to what extent the mechanistic insight we have derived from the ZBP1 KH3-KH4 model is applicable to other RNA-binding di-domains (e.g., the ones of the hnRNPE1, E1, and K proteins), in which the K_d_ of individual domains for the RNA targets is also in the low μM range and RNA looping has been proposed to be an important component of the recognition mechanism.

A key objective of this study is to understand how the ZBP1-*β-actin* interaction is regulated by the cellular concentration of protein and RNA, and to extend this initial model to interpret data on other ZBP1 targets that share a similar KH3-KH4-based interaction mode. The concentration of ZBP1 in the cell is sub-micromolar, which is nearly three orders of magnitude higher than the *β-actin* RNA concentration. Our model indicates that at these protein and RNA concentrations, binding is dependent on the concentration of the protein, but not on the concentration of the RNA. This conclusion would hold even if the RNA concentration was 50-fold higher, for example, because of higher local concentration, or 50-fold lower than the one used here. *β-actin* is a housekeeping gene, and cellular levels of *β-actin* mRNA are high and maintained in tissues and cancers (https://genevisible.com/cancers/HS/UniProt/P60709). ZBP1, on the other hand, is expressed at high levels at a defined stage in neuronal development, but is low in many adult tissues, and we propose that this mechanism allows the protein-RNA interaction to be regulated effectively by varying the protein concentration within a defined time window. Interestingly, many ZBP1 targets are not housekeeping genes, and their concentrations show a many-fold variation during development and cell cycle (Conway et al., 2016). Our model indicates that as long as the overall concentration of available RNA targets is significantly lower than the protein concentration and the targets have a similar affinity and binding mode, ZBP1 can effectively regulate the targets independently of their expression levels. Although additional layers of regulation likely control ZBP1 binding in a target-specific fashion, the concept highlighted above may help de-convolute this complexity in ZBP1 and similar systems, in which a protein recognizes a common RNA recognition element with high specificity.

This study provides a mechanistic framework for quantitatively interpreting a diverse range of in-cell and genomic observations on the ZBP1-*β-actin* interaction and identifies key parameters for its regulation. It is worth mentioning that the sub-micromolar ZBP1 concentration that was recently measured in mouse embryonic fibroblasts is close to the previously estimated level of ZBP1 expression in a human cancer cell line (∼400,000 molecules per cell) (Liao et al., 2004), suggesting that our conclusions on the regulation of ZBP1-RNA interaction are not limited to developmental neurons but are relevant to the role of ZBP1 in promoting tumor metastasis.

## Experimental Procedures

### Protein and RNA Sample Preparation

The KH3-KH4 di-domain construct (P386-G569, Y396F) of *G. gallus* Zipcode binding protein 1 (accession number AF026527) and its GxxG-GDDG mutants were cloned, expressed, and purified as previously described (Hollingworth et al., 2012). Briefly, the proteins were expressed as fusion proteins in *E. coli* BLI21 (DE3) cells (Invitrogen) and purified using an IMAC column. The affinity tag was then cleaved off, and the wanted protein construct was further purified using a MonoQ 5/50 GL column. Protein purity (>95%) and integrity were confirmed using SDS-PAGE and electrospray mass spectrometry, and the protein was then stored at −20°C in 20 mM NaPi, pH 6.5, 20 mM NaCl, 0,05% NaN3, and protease inhibitors (Roche). Concentrations were determined using absorption spectroscopy. Unlabeled samples were obtained from protein expressed in LB media, whereas samples labeled with NMR-active, stable isotopes (different combinations of ^2^H, ^15^N, and ^13^C) were obtained using labeled media as described (Cukier et al., 2010). RNA oligonucleotides were purchased from Dharmacon and Integrated DNA Technologies, de-protected by following the manufacturer’s instructions, lyophilized, and resolubilized in the appropriate buffer. RNA concentrations were calculated using absorption spectroscopy.

### NMR Spectroscopy

NMR experiments were recorded at temperatures between 25°C and 37°C on Bruker Avance and Varian Inova spectrometers operating at a 700-, 600-, and 800-MHz ^1^H frequency. NMR spectra were processed by using the NMRpipe suite of programs (Delaglio et al., 1995) and analyzed by using the Sparky (Pettersen et al., 2004) and XEASY (Bartels et al., 1995) programs. Protein and RNA samples were in a 10% D_2_O 90% H_2_O solution of 20 mM phosphate buffer and 20 mM NaCl at pH 6.5. Protein backbone and side-chain resonance assignments of the two RNA oligonucleotides were obtained as previously described (Nicastro et al., 2012) and detailed in the Supplemental Experimental Procedures section. ^15^N T_1_ and T_2_ values and ^15^N heteronuclear NOE values were obtained from standard experiments recorded at 600-MHz proton as described (Kay et al., 1989). Intermolecular NOEs were obtained from 2D ^1^H-^1^H NOESY spectra, 3D ^15^N NOESY-HSQC, 3D ^13^CNOESY-HSQC, as well as 3D-filtered ^13^CNOESY, with ^13^C and ^15^N rejected (150-ms mixing time) recorded on 1:1 protein (labeled):RNA (unlabeled) samples.

### Structure Calculations

The structures of the KH3-GCACACCC and KH4-UCGGACU complexes were calculated using a semi-automated ARIA-2.3-based protocol (Linge et al., 2003) and refined in a shell of explicit water as described. Hydrogen bond restraints were added only in the final set of calculations if a proton was hydrogen bonded in at least 50% of the initial set of structures, as detailed in the Supplemental Experimental Procedures section. Structural statistics were computed for the final ensemble of 12 deposited structures using PSVS 1.5. Structures were analyzed visually using the program Pymol, which was also used for all graphical representations (The PyMOL Molecular Graphics System, Version 1.8, Schrödinger). The modeling of the U nucleobase in the KH4 RNA-binding groove was executed with the program Insight2 (Accelrys).

### ITC

ITC experiments were recorded on a VP-ITC or MicroCal iTC200 instrument (GE Healthcare) at 25°C. Protein and RNA samples were dialysed in 20 mM NaPi, pH 6.5, and 100 mM NaCl. For all samples, small aliquots of a 200–300 μM protein solution were injected into a cell containing a 10–15 μM RNA solution, and the heat of reaction was measured. Data were analyzed using Microcal Origin 7.0 software. The independently measured heat of dilution was subtracted, and the dissociation constants (K_d_) were obtained by fitting the data with a one (for the short RNA sequences) or two non-sequential (for the full-length Zipcode) binding-site model.

### BLI

BLI experiments were performed on a ForteBio OctetRed instrument. Biotinylated RNAs were acquired from Dharmacon and dissolved in 0.22-μm filtered buffer (20 mM phosphate, pH 6.5, and 100 mM NaCl) containing 2 mM TCEP, 2 mg/mL of bovine serum albumin (BSA), and 0.005% Tween 20 to reduce non-specific interactions. The assays were carried out at 25°C in a 96-well plate and a sample volume of 200 μL. Streptavidin-coated biosensors were pre-equilibrated, loaded with biotinylated RNAs (ranging from 144 ng/mL to 432 ng/mL in assay buffer), and exposed to protein concentrations ranging from 5 to 160 nM (for KH3-KH4) and from 125 to 8,000 nM (for the two protein mutants). Data were processed, and kinetic parameters were calculated using ForteBio or in-house software (Martin et al., 2000). The k_on_ values were obtained as the slope of a plot of observed association rate constant against protein concentration. The K_d_s were determined by fitting the maximum response values measured as a function of protein concentration. The k_off_ values were obtained from k_off_ = K_d_ × k_on_ (Cukier et al., 2010). The accuracy of the k_off_ values was confirmed from direct analysis of the dissociation phase.

### Simulations

The simulation was performed by numerical integration of the system of ordinary differential equations associated with the model presented here. In-house software used the fourth-order Runge-Kutta method as described by Press et al. (2007). The computer code is available upon request.

## Author Contributions

Original Clones, D.H.; Samples Preparation, A.M.C., and, in a few cases, by D.H. and G.N.; NMR experiments, G.N., A.M.C., and A.O.; KH3-KH4 Assignment, A.M.C. and G.N.; Structure of the KH3-Bound Complex, A.M.C. and G.N.; Structure of the KH4-Bound Complex, G.N.; ITC, G.N.; BLI, performed by G.N. and analyzed by G.N. and S.R.M.; Biophysical Modeling, S.R.M.; Bioinformatics, M.U. and R.B. The project was designed by A.R.; The paper was written by A.R., S.R.M., and G.N. with M.U. and R.B., and revised by all the authors.

## Figures and Tables

**Table 1:** NMR and Refinement Statistics for Complexes

	ZBP1-KH3KH4DD Protein	CACACCC RNA	ZBP1-KH3DDKH4 Protein	UCGGACU RNA
NMR distance and dihedral constraints				
Distance restraints				
Total NOE	2,404	52	2,333	39
Intra-residue	1,261	35	1,236	15
Inter-residue	1,143		1,097	
Sequential (|*i* – *j*| = 1)	474	17	427	24
Nonsequential (|*i* – *j*| > 1)	669		663	
Hydrogen bonds				
Protein-nucleic acid intermolecular	28		33	
Total dihedral angle restraints				
Protein	205		205	
ϕ	103		103	
ψ	102		102	
Nucleic acid				
Sugar pucker		7		5
Backbone		19		17
Structure statistics				
Violations (mean and SD)				
Distance constraints (Å) (>0.3 Å)	3		1	
Dihedral angle constraints (°)	0		0	
Maximum dihedral angle violation (°)	0		0	
Maximum distance constraint violation (Å)	0.387 ± 0.029		0.325 ± 0.014	
Deviations from idealized geometry				
Bond lengths (Å)	0.002 ± 0.001		0.002 ± 0.001	
Bond angles (°)	0.383 ± 0.097		0.340 ± 0.012	
Impropers (°)	0.316 ± 0.220		0.229 ± 0.020	
Average pairwise root-mean-square deviation (RMSD)^a^ (Å)				
Protein				
Heavy	1.3 ± 0.14		1.4 ± 0.15	
Backbone	0.9 ± 0.11		0.9 ± 0.10	
RNA				
All RNA heavy		0.50 ± 0.17		0.78 ± 0.16
Complex				
All complex heavy (C, N, O, P)	1.55 ± 0.13		1.71 ± 0.18	

a Structural statistics were computed for ensembles of 12 deposited structures using PSVS 1.5. Ordered residues ([S(phi) + S(psi) > 1.8]): KH3: 405–422, 425–479, 482–503, 509–525, and 529–565; KH4: 406–422, 425–443, 450–479,485–503, 508–523, and 529–565.

## Accession Numbers

The accession numbers for the atomic coordinates for the NMR structure ensembles and the corresponding chemical shift assignment are PDB: 2N8L and 2N8M and the Biological Magnetic Resonance Databank: 25854 and 25855.

## Supplemental Information

Document S1. Supplemental Experimental Procedures, Figures S1–S5, and Tables S1 and S2

Document S2. Article plus Supplemental Information
