# Supplementary material for: Mechanism of β-actin mRNA Recognition by ZBP1
Source: Cell Rep. 2017 Jan 31;18(5):1187–99. doi: 10.1016/j.celrep.2016.12.091 (PMC5300891; doi:10.1016/j.celrep.2016.12.091)
Supplement: Document S1. Supplemental Experimental Procedures, Figures S1–S5, and Tables S1 and S2 [file mmc1.pdf]

Cell Reports, Volume *18*

Supplemental Information

Mechanism of β-actin mRNA Recognition by ZBP1

Giuseppe Nicastro, Adela M. Candel, Michael Uhl, Alain Oregioni, David Hollingworth, Rolf Backofen, Stephen R. Martin, and Andres Ramos

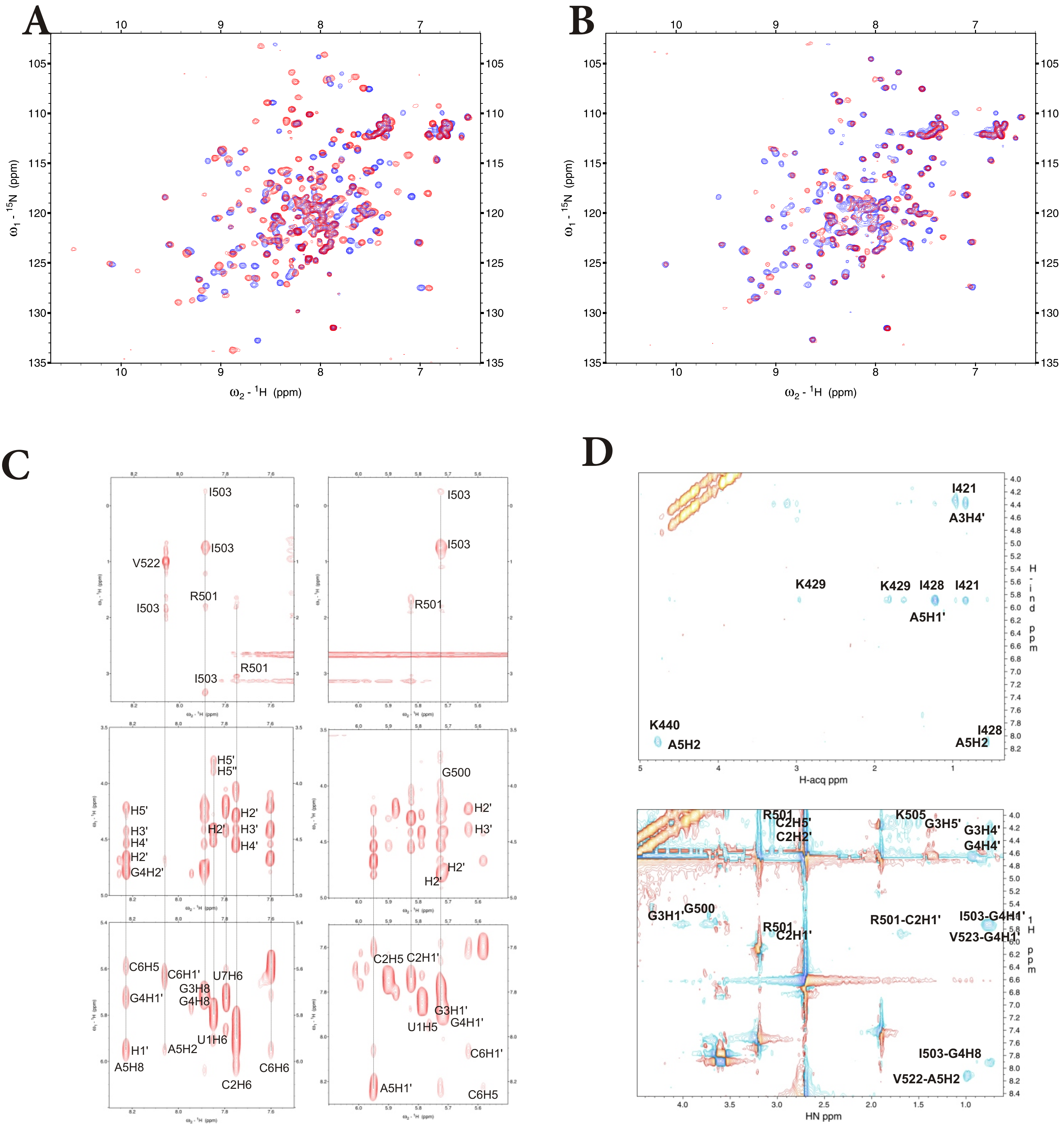

Figure S1 - NMR of the ZBP1 KH3-KH4-RNA complexes, related to Figures 2 and 3. (A) Overlay of free (blue) and bound (red) ^{15}N HSQC spectra of KH3-KH4DD (KH3 binding)-RNA complex (B) Overlay of free (blue) and bound(red) ^{15}N HSQC spectra of KH3DD-KH4 (KH4 binding)-RNA complex (C) Homonuclear ^{1}H-^{1}H spectrum
(250ms mixing time) recorded on a sample of KH3DD-KH4 (KH4 binding)-RNA complex. Representative intermolecular NOE cross-peaks are labelled. Some intra-molecular NOEs are also labelled for comparison. (D) 2D-(^{1}H-^{1}H) F1-^{13}C filtered, F2-^{13}C edited NOESYs recorded on samples of KH3-KH4DD (KH3 binding)-RNA and KH3DD-KH4 (KH4 binding)-RNA complexes. Representative NOEs cross-peaks are labelled. Some intra-molecular NOEs are also labelled for comparison.

Figure S1

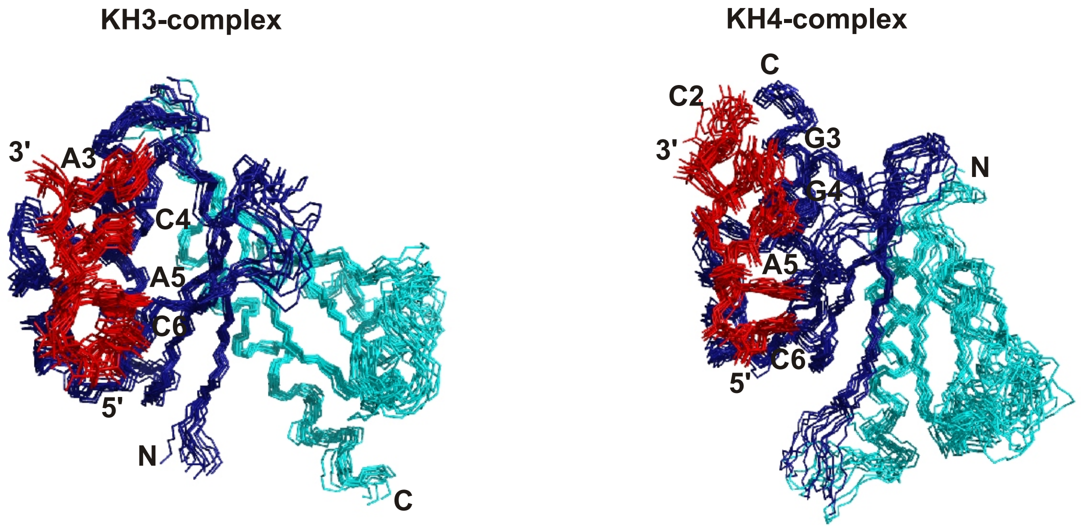

Figure S2 - NMR structures bundle. Superimposition of the 12 lowest energy solution structures of ZBP1 KH3-KH4DD-RNA complex (left) and ZBP1 KH3DD-KH4-RNA complex (right), related to Figures 2 and 3. The protein backbone is in blue (with the interacting domain in dark blue) and the RNA backbone in red. The variable loop of KH4 is less well defined than for KH3, consistently with the dynamics observed in the variable loop (Figure S4).

Figure S2

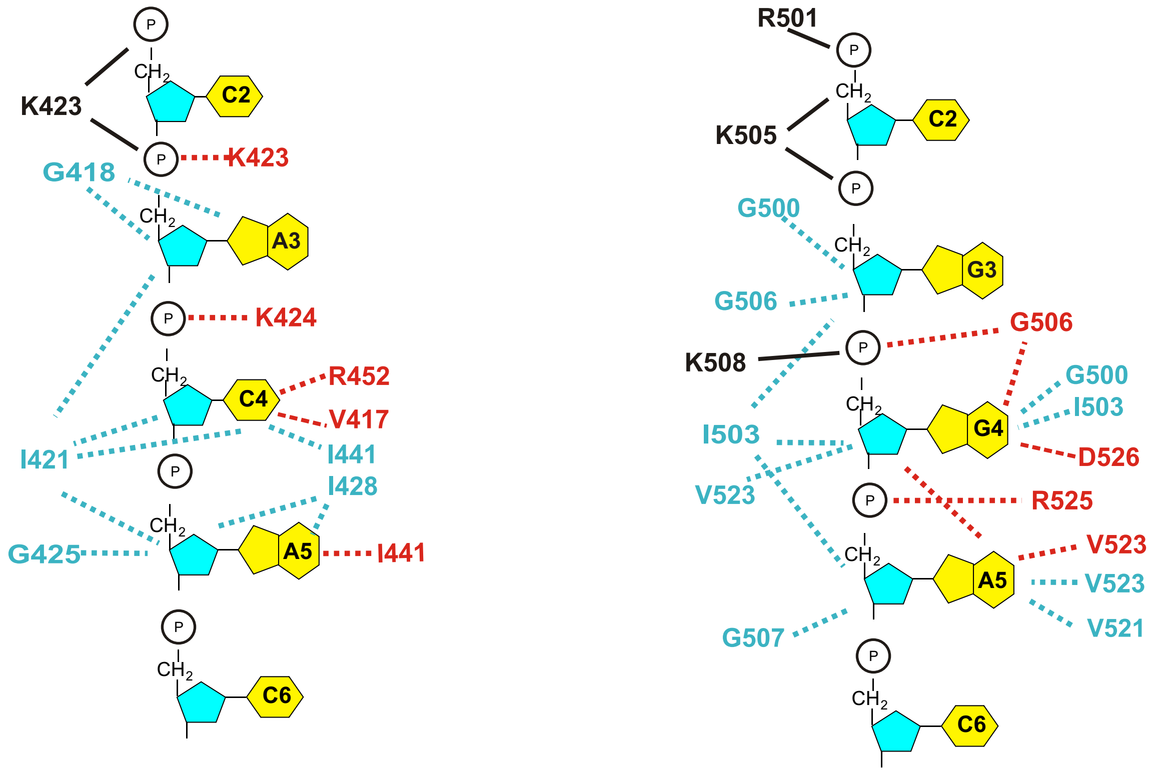

Figure S3 - Protein-RNA contacts in the KH3-RNA and KH4-RNA binding complexes, related to Figures 2 and 3. Protein-RNA contacts observed in KH3-KH4DD-RNA (KH3 binding, left) and KH3DD-KH4-RNA (KH4 binding, right) complexes. Hydrogen bonds are in red, hydrophobic interactions are in blue and electrostatic interactions involving phosphate group are represented by a black line. Contacts observed in the majority of structures in the bundle are reported.

Figure S3

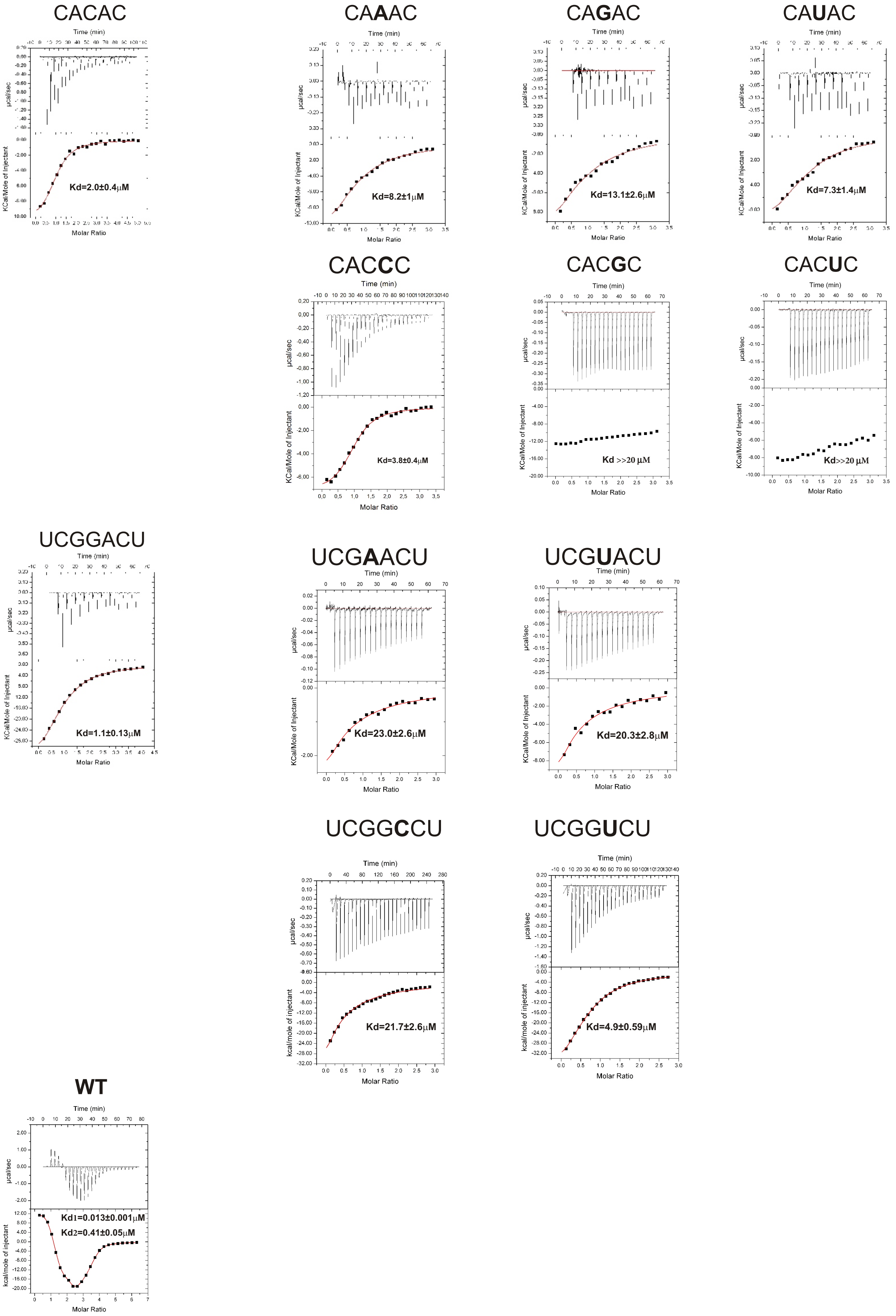

CACAC
Kd=2.0±0.4μM
CAAAC
Kd=8.2±1μM
CAGAC
Kd=13.1±2.6μM
CAUAC
Kd=7.3±1.4μM
CACCC
Kd=3.8±0.4μM
CACGC
Kd >>20 μM
CACUC
Kd>>20 μM
UCGGACU
Kd=1.1±0.13μM
UCGAACU
Kd=23.0±2.6μM
UCGUACU
Kd=20.3±2.8μM
UCGGCCU
Kd=21.7±2.6μM
UCGGUCU
Kd=4.9±0.59μM
WT
Kd1=0.013±0.001μM
Kd2=0.41±0.05μM
Time (min)
μcal/sec
KCal/Mole of Injectant
Molar Ratio

Figure S4

Figure S4 - Nucleobase preference of KH3 and KH4. ITC titrations of the KH3-KH4DD and KH3DD-KH4 proteins with the target RNA sequences and with single nucleotide mutations, related to Figures 2 and 3. Experiments were performed at 25ºC in 20 mM phosphate buffer pH 6.5 and 100mM NaCl. Top – baseline corrected calorimetric titration data. Bottom - binding isotherm derived by integrating the area of each peak after each injection. The solid line represents the best fit of the data to a single binding site model. Kd values are reported on each plot. Also an equivalent titration was performed on the KH3-KH4 (bottom) protein with the 28 nucleotides Zipcode RNA. Here two binding events are visible, the specific high affinity binding with a Kd of approximately 13 nM.

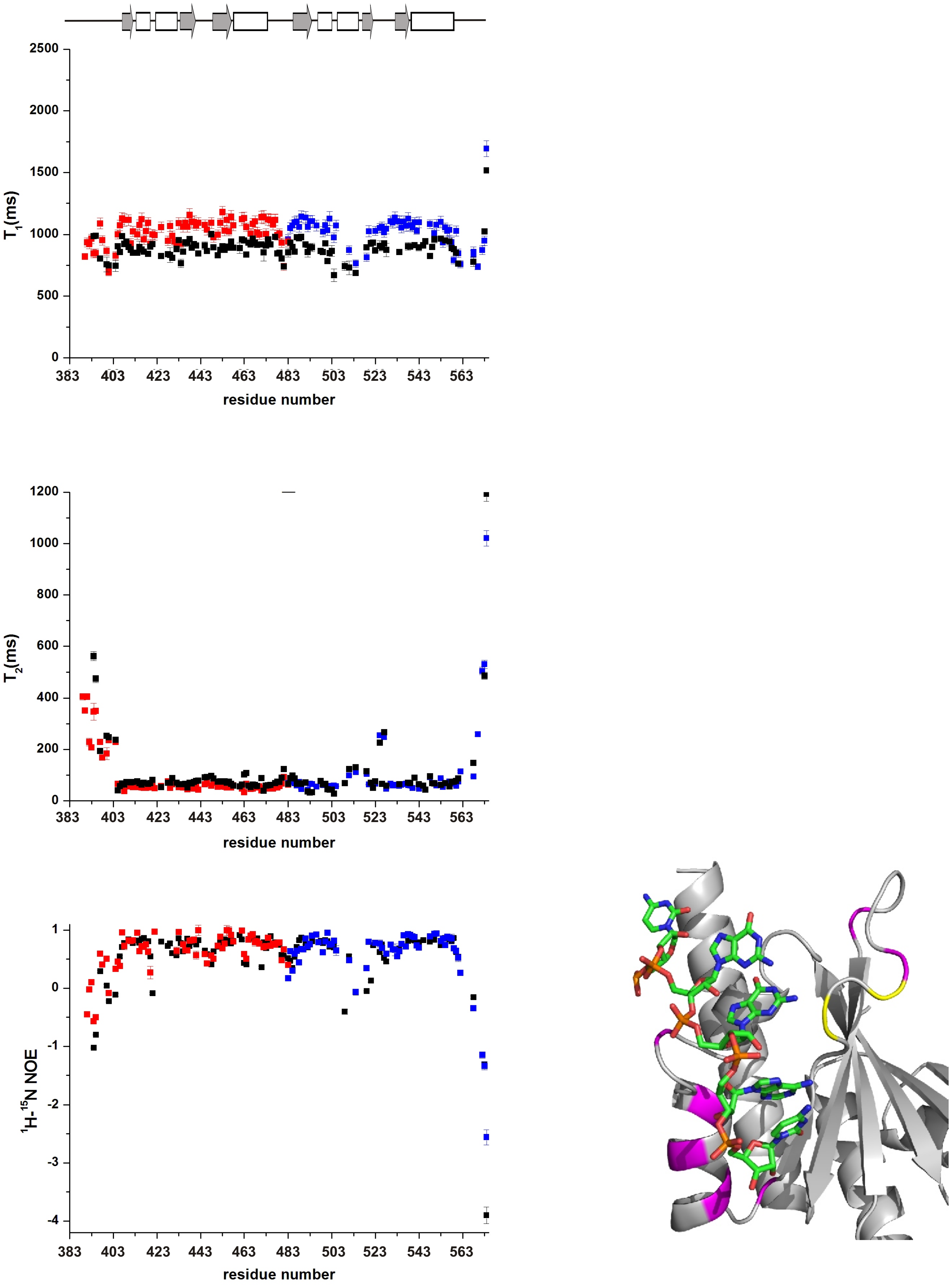
2500
2000
1500
1000
500
0
T1(ms)
383
403
423
443
463
483
503
523
543
563
residue number
1200
1000
800
600
400
200
0
T2(ms)
1
0
-1
-2
-3
-4
1H-15N NOE

Figure S5

Figure S5 - KH3-KH4 backbone dynamics and changes upon RNA binding, related to Figure 4.
Left - Backbone relaxation of free and bound KH3-KH4. ^{15}N T1 (top), ^{15}N T2 (middle) and $^{15}N\{^1H\}$ heteronuclear NOE (bottom) values are plotted against residue number. Free protein values are in black. KH3-bound values are in red and KH4-bound values are in blue. For simplicity, only the values of the bound domains in the two-domain complexes (KH3 in one case and KH4 in the other) are displayed, as the trend of values of the unbound domains is the same as in the free protein. Changes in the overall T1 and T2 values are consistent with the small increase in molecular weight upon RNA binding. Secondary structure elements are displayed above. Right – the residues of the KH4 domain with heteronuclear NOE values of <0.6 (magenta, high frequency motions) and with T2 values of > 200 ms (yellow, low frequency motions) are colored on the backbone ribbon representation of the bound KH4 which is otherwise in grey).

Table S1

Change in the population of the bound RNA species in function of kC3, related to Figure 6

kC3	kC4	Free[RNA]	[Protein]	Total RNA bound	RNA bound as C	RNA % bound as C	Kd	koff
0.1	0.46666	2.17E-10	2.00E-07	1.83E-10	1.02E-10	4.57E+01	2.38E-07	2.92E-02
0.2	0.933333	1.73E-10	2.00E-07	2.27E-10	1.62E-10	5.67E+01	1.53E-07	2.01E-02
0.5	2.333333	1.08E-10	2.00E-07	2.92E-10	2.52E-10	7.31E+01	7.38E-08	1.09E-02
1	4.66666	6.61E-11	2.00E-07	3.34E-10	3.09E-10	8.35E+01	3.96E-08	6.25E-03
1.5	6.999999	4.77E-11	2.00E-07	3.52E-10	3.35E-10	8.81E+01	2.71E-08	4.41E-03
2	9.33333	3.73E-11	2.00E-07	3.63E-10	3.49E-10	9.07E+01	2.06E-08	3.42E-03
3	14	2.60E-11	2.00E-07	3.74E-10	3.64E-10	9.35E+01	1.39E-08	2.37E-03
4	18.66666	1.99E-11	2.00E-07	3.80E-10	3.73E-10	9.50E+01	1.05E-08	1.82E-03
6	28	1.36E-11	2.00E-07	3.86E-10	3.81E-10	9.66E+01	7.04E-09	1.25E-03
8	37.3333	1.03E-11	2.00E-07	3.90E-10	3.86E-10	9.74E+01	5.30E-09	9.64E-04
10	46.66666	8.32E-12	2.00E-07	3.92E-10	3.89E-10	9.79E+01	4.25E-09	7.91E-04

Total [RNA] = 4E -10
RNA bound as C = RNA bound in the closed complex
RNA % bound as C = percentage of the total bound RNA present in the closed complex form

Table S2

Variation in the amount of bound RNA in function of ZBP1 concentration, related to Figure 6

[Protein]	[RNA]	Total RNA bound	RNA bound as C	% RNA bound as C
5.00E-09	3.23E-10	7.72E-11	7.44E-11	1.93E+01
1.00E-08	2.70E-10	1.30E-10	1.25E-10	3.24E+01
2.50E-08	1.82E-10	2.19E-10	2.11E-10	5.46E+01
5.00E-08	1.17E-10	2.83E-10	2.73E-10	7.07E+01
1.00E-07	6.84E-11	3.32E-10	3.19E-10	8.29E+01
2.00E-07	3.73E-11	3.63E-10	3.49E-10	9.07E+01
4.00E-07	1.95E-11	3.81E-10	3.65E-10	9.51E+01
6.00E-07	1.32E-11	3.87E-10	3.70E-10	9.67E+01

Total [RNA] 4E -10

kC3=2

kC4=9.3333

Supplemental Experimental Procedures

Resonance assignment and structure calculations

Protein backbone and side-chain resonance assignments were obtained from 2D ^{1}H-^{15}N HSQC, 2D ^{1}H-^{13}C HSQC, 3D HNCA, 3D CBCACONH, 3D HNCACB, 3D [^{1}H-^{13}C-^{1}H] HCCH-TOCSY, [^{13}C-^{13}C-^{1}H] HCCH-TOCSY, 3D ^{15}N NOESY-HSQC, 3D ^{13}C-NOESY-HSQC experiments, optimized for either aliphatic and aromatic resonances, as previously described (Nicastro et al., 2012). Resonance assignments of the two RNA oligonucleotides, free and in complex were obtained from 2D ^{1}H-^{1}H TOCSY, 2D ^{1}H-^{1}H NOESY either decoupled or un-decoupled. NOESY spectra were recorded using mixing times of 100, 150 and 250ms depending on the sample. TOCSY spectra were recorded using a mixing time of 60ms. Intramolecular NOEs were obtained from 3D ^{15}N NOESY-HSQC, 3D ^{13}C-NOESY-HSQC experiments. Inter-molecular NOEs were obtained as described in the methods section.

The structures of the KH3–GCACACCC and KH4-UCGGACU complexes were calculated using the protocol described (Nicastro et al., 2012) with a few variations as described below. Briefly, the structures were calculated using a semi-automated ARIA 2.3–based protocol (Linge et al., 2003). Distance restraints were obtained by integrating NOE cross-peaks in 3D and 2D NOESY spectra using the XEASY program (Bartels et al., 1995). The protein-protein NOE cross-peaks were calibrated automatically and assigned iteratively within ARIA, whereas the peaks arising from RNA proton resonances were calibrated manually in a semi-quantitative fashion (Varani et al., 1996). Protein angle restraints were obtained from the chemical shifts of CO, CA, CB and backbone amide N and NH moieties using the program TALOS. RNA angle restraints (α, ζ and δ) were obtained from ^{1}H-^{1}H TOCSY spectra and ^{31}P-^{1}H correlation spectra. Hydrogen bond restraints were added only in the final set of calculations and only in well-defined secondary structure elements if a proton was hydrogen-bonded in at least 50% of the initial set of structures. Further, the structures of both the KH3 and the KH4 complex show the canonical double H-bond between the nucleobase in position 3 and the protein backbone in the initial set of structures and these hydrogen bonds were added at the last step of the calculations.

ARIA 2.3 was used to calculate 100 conformers of the complex (iterations 0–7). The 20 conformers with the lowest restraint energies were refined in a shell of explicit water. The 12 conformers with the lowest restraint energies, restraint violations and r.m.s. deviations from the ideal covalent geometry were taken as representative of the converged structures and selected for structural analysis. Structural statistics were computed for ensemble of 12 deposited structures using PSVS 1.5. The Ramachandran plot analysis for the two families of structures showed that for ordered residues ([S(phi)+S(psi)> 1.8]): 405-422, 425-479, 482-503, 509-525, 529-565 of KH3, 95.5% are in the most favored, 4.4 % additional, 0.1% generously and 0% disallowed regions respectively, and for ordered residues of KH4 (406-422, 425-443, 450-479, 485-503, 508-523, 529-565) 93.5% are in the most favored, 6.5% additional, 0% generously and 0.1% disallowed regions respectively.

Calculation of kC3 and kC4

To calculate kC3, we consider the pathway for formation of the closed complex in which the KH4 domain attaches first (see Figure 5). This pathway is effectively a simple reversible bimolecular reaction followed by a conformational change that can be summarized as:

$$P + R \underset{k_1}{\overset{k_1}{\rightleftarrows}} PR \underset{k_2}{\overset{k_2}{\rightleftarrows}} PR^*$$

For a reaction of this type the experimentally measured equilibrium dissociation constant (K_d) is related to the individual constants (K_{d1} and K_{d2}) by $Kd = K_{d1}K_{d2}/(1+K_{d2})$

(Eccleston et al., 2008). For the purpose of calculating kC3, the reaction can be written as:

$$P + R \underset{0.13}{\overset{1.4x10^5}{\rightleftarrows}} PR \underset{kO3}{\overset{kC3}{\rightleftarrows}} PR^*$$

Therefore in our case $K_{d1} = 0.13/1.4x10^5 = 9.286 \text{ x } 10^{-7}$ M and K_{d2} = kO3/kC3.. Since the experimentally measured K_d is $2.06 \text{ x } 10^{-8}$ M one has

$2.06 \text{ x } 10^{-8} = 9.286\text{x } 10^{-7} \text{ x } K_{d2}/(1+K_{d2})$

$0.0222 = K_{d2}/(1+K_{d2})$ and therefore $K_{d2} = 0.0227$

If we assume for the sake of simplicity that the ring opening step (kO3) in which KH3 dissociates to re-form the 1:1 complex has the same rate constant as that for dissociation from the appropriate 1:1 complex then kO3 = 0.046 s^{-1}.

Then K_{d2} = 0.0227 = 0.046/kC3 and kC3 = ~ 2 s^{-1}

The same calculation for the other pathway would mean that kC4 was equal to ~ 9.3 s^{-1}.

Supplemental references

Bartels, C., Xia, T., Billeter, M., Guntert, P., and Wuthrich, K. (1995). The program XEASY for computer supported NMR spectral analysis of biological macromolecules. J. Biomol. NMR 6, 1–10.

Eccleston JF, Martin SR, Schilstra MJ (2008) Rapid kinetic techniques. Meth. Cell Biol. 84, 445-477.

Kay, L.E., Torchia, D.A. and Bax, A. (1989). Backbone dynamics of proteins as studied by 15N inverse detected heteronuclear NMR spectroscopy: application to staphylococcal nuclease. Biochemistry 28, 8972-8979.

Linge, J.P., Habeck, M., Rieping, W., and Nilges, M. (2003). ARIA: automated NOE assignment and NMR structure calculation. Bioinformatics 19, 315–316.

Nicastro, G., García-Mayoral, M.F., Hollingworth, D., Kelly, G., Martin, S.R., Briata, P., Gherzi, R., and Ramos, A. (2012). Noncanonical G recognition mediates KSRP regulation of let-7 biogenesis. Nat. Struct. Mol. Biol. 19, 1282-1286.

Varani, G., Fareed, Aboul-ela., & Allain, Frederic-H-T. NMR investigation of RNA structure Prog. Nucl. Magn. Reson. Spectrosc. 29, 51–127 (1996).
